# Electronic, heat-not-burn, and combustible cigarette use among chronic disease patients in Japan: A cross-sectional study

**DOI:** 10.18332/tid/94455

**Published:** 2018-09-10

**Authors:** Yoshiyuki Kioi, Takahiro Tabuchi

**Affiliations:** 1 School of Medicine, Osaka University, Osaka, Japan; 2 Osaka International Cancer Institute, Osaka, Japan

**Keywords:** electronic cigarettes, heat-not-burn tobacco, chronic disease patients, atopic dermatitis, mental disorders

## Abstract

**INTRODUCTION:**

Although tobacco smoking adversely affects health, many people continue to smoke while suffering from chronic disease. Few studies have examined electronic and heat-not-burn cigarette use among chronic disease patients. Our objective was to investigate electronic, heat-not-burn and combustible cigarette use among chronic disease patients with hypertension, diabetes, cerebrovascular disease, COPD (chronic obstructive pulmonary disease), asthma, atopic dermatitis, cancer, or mental disorders.

**METHODS:**

We analyzed 4432 eligible respondents aged 40–69 years from a 2015 internet survey (randomly sampled research agency panelists) with a propensity score weighting adjustment for ‘being a respondent in an internet survey’ in Japan. The outcome measure was the prevalence of electronic, heat-not-burn, and combustible cigarette use. Intention to quit was also calculated.

**RESULTS:**

In all, 32.1% (n=80) of male patients with more than two diseases and 10.3% (n=16) of female patients were current smokers. Of the patients who had no intention to quit smoking, 15.5% were male patients with atopic dermatitis and 63.7% with mental disorders. Of the men, 7.7% without any diseases had ever used e-cigarettes, while 7.7% and 6.4% of men with one disease or more than two diseases, respectively, had ever used e-cigarettes. Of the women, 3.5% without any diseases had ever used e-cigarettes, while 2.1% and 2.9% of women with one disease or more than two diseases, respectively, had ever used e-cigarettes. Percentage of heat-not-burn tobacco current or ever use was low (<0.1%) among both men and women.

**CONCLUSIONS:**

Differences in the use of electronic and combustible cigarettes according to the number of diseases were not obvious. However, sex differences for smoking among chronic disease patients, especially in atopic dermatitis and mental disorders, were found.

## INTRODUCTION

Many chronic disease sufferers continue to smoke despite the adverse effects of smoking on health. Continued smoking after a cancer diagnosis may adversely affect treatment effectiveness, subsequent cancer risk, and survival^1^. However, about 36.1% of adult cancer survivors who were smoking regularly at the time of their cancer diagnosis continued to smoke after diagnosis^2,3^. Another report showed that only 4.7% of colorectal cancer patients who smoked at the time of the cancer diagnosis stopped smoking after diagnosis^4^. Of the cancer patients who continued to smoke after diagnosis, 70% were willing to reduce or quit smoking, but in most cases they were unable to stop smoking^5^. Previous studies that examined patients with diseases other than cancer are scarce^2-4,6^.

Recently, electronic cigarettes (e-cigarettes) have gained popularity worldwide. In Japan, 48% were aware of e-cigarettes, and smokers showed 20–30% more recognition of e-cigarettes than non-smokers in 2015^7^. To date, few studies have examined e-cigarette use among chronic disease patients^6,8,9^. In South Korea, 3.9% of patients with asthma had used e-cigarettes at least once in the last month^8^. In the US, an average 9.9% of cancer patients used e-cigarettes, ranging from 5.6% of patients with gynecological cancer to 23.5% of those with urology cancer^9^. Recently, the relationship between chronic diseases (asthma, COPD, cancer, cardiovascular disease, stroke, hypertension, diabetes) and e-cigarette use was reported in the US^6^. However, e-cigarette use among mental disorder patients has not been fully elucidated^6,8-10^.

The number of diseases, or a specific disease, may affect the usage of electronic, heat-not-burn and combustible cigarettes. In this study, we analyzed: 1) combustible tobacco use and intention to quit smoking, and 2) electronic and heat-not-burn tobacco use; among patients with various diseases including mental disorders.

## METHODS

### Setting and sample

An internet survey was conducted between 31 January and 17 February 2015 among the first 9000 respondents7; i.e. 500 persons aged 15–19 years and 800 persons aged 20–29, 30–39, 40–49, 50–59 and 60–69 years, for both sexes. Adjusted basic characteristics of study subjects and median age with range of each group are shown in Tables S1 and S2 (Supplementary file, see Appendix), respectively. We used data from 2193 men and 2239 women aged 40–69 years because the prevalence of chronic diseases was low in young people (for reference, estimates among young people aged 15–39 years are shown in Table S3).

### Procedures

Participants were invited from a large survey panel managed by a major nationwide internet research agency, Rakuten Research^7^. The overall size of the survey panel at the time of the survey was about 2.3 million people, of whom 53.9% were male^7^. At the time of registration, panel participants were required to provide information on their sex, age, occupation and residence, and to agree that they would participate in different research surveys.

In this survey, panelists were asked about their use of combustible tobacco and e-cigarettes and any health-related factors, such as diagnosis or hospital visits for chronic diseases. The survey requests were sent by the research agency to the panelists who were selected for each sex and age category using simple random sampling. Panelists who consented to participate in the survey accessed the designated website and responded to the survey. The survey was closed when the target numbers of respondents for each sex and age category were met. The participation rate^11^ for the survey was 8.5% (9055/106202). Detailed information for the survey was available from a previous study^7^. The study protocol was approved by the Research Ethics Committee of the Osaka Medical Center for Cancer and Cardiovascular Diseases in 2014 (No. 1412175183).

### Combustible cigarette use and intention to quit smoking

Panelists were asked: ‘Please choose your current status for combustible cigarette (boxed cigarette or roll-your-own cigarette)’; and the response options were ‘never user’, ‘former non-regular user’, ‘former regular user’ and ‘current user’. Respondents who currently smoked combustible cigarette (boxed cigarette and/or roll-your-own cigarette) were considered current smokers. Those who reported former use and did not currently smoke either type of cigarette were considered former smokers. Those who had never smoked were considered never smokers.

Panelists were also asked: ‘Please choose your current intention to quit smoking’; and the response options were ‘I have no intention to quit smoking’, ‘I want to quit smoking more than 6 months from now’, ‘I want to quit smoking between 1 month and 6 months’ and ‘I want to quit smoking within 1 month’. The first two categories were combined and defined as no intention to quit.

### Electronic and heat-not-burn cigarette use

Recently, multiple types of electronic and heat-not-burn cigarettes have become available in the Japanese market. For example, a new heat-not-burn cigarette ‘Ploom’ has been sold by Japan Tobacco (JT) since December 2013. Also, a new heat-not-burn cigarette ‘iQOS’ has been sold by Philip Morris (PM) since November 2014. Panelists were asked about their use of each of the following products: nicotine e-cigarettes, non-nicotine e-cigarettes, e-cigarettes with unknown nicotine content, Ploom and iQOS, using the question: ‘Please choose your current status for each product’; and the response options were ‘never user’, ‘former non-regular user’, ‘former regular user’ and ‘current user’. The last three responses were combined and defined as ‘ever user’ of each product. Respondents who reported ever-use (at least once) of at least one type of e-cigarette were considered e-cigarette ever-users. Respondents who reported ever-use of Ploom or iQOS were heat-not-burn tobacco ever-users. Those who reported a number greater than zero for the question ‘During the past 30 days, on how many days have you used e-cigarettes or heat-not-burn cigarettes?’ were defined as e-cigarettes or heat-not-burn cigarettes user in the last 30 days^7,12,13^.

### Chronic diseases

Panelists were asked about their health status, using the question: ‘Do you have any of the following chronic diseases (for which you have received a diagnosis or are regularly visiting hospital)—hypertension, diabetes, cerebrovascular disease, asthma, atopic dermatitis, COPD, cancer, or mental disorders?’; and the response options were ‘never affected’, ‘formerly affected’, ‘currently affected or regularly visiting hospital (clinic)’ and ‘currently affected and without ambulant treatment’. The last two responses were combined and defined as ‘currently affected’ for each disease. Diabetes, asthma, cerebrovascular diseases, COPD and cancer were combined and defined as ‘tobacco-related diseases’^14,15^.

### Statistical analyses

All statistical analyses were performed using EZR (Easy R) version 1.33 (constructed by Kanda Y., Saitama Medical Center Jichi Medical University, JAPAN^16^) and SAS version 9.3 (SAS Institute Inc., Cary, NC, US). Survey weights were used for generalizability, and weighted results are shown. Women and men were analyzed separately. A Fisher exact test was used to compare the differences between people who have no diseases (healthy reference) and patients with chronic disease.

Although internet surveys have several advantages compared to traditional surveys, their greatest potential drawback is that they may not be representative of the general population because specific type of people may only access the internet. Previous studies have suggested that the adjusted estimates using inverse probability weighting (IPW) obtained from a propensity score (calculated by logistic regression models using basic demographic and socioeconomic factors, such as education and housing tenure) from an internet-based convenience sample provide similar estimates of parameters, or at least reduced the differences compared to probability sample based estimates^17-19^. Therefore, IPW-adjusted estimates rather than simple internet survey estimates are presented as the main results in this study (unadjusted estimates are shown in Table S4). To correct for the selectivity of internet-based samples, we used a probability sample that is representative of the Japanese population from the Comprehensive Survey of Living Conditions of People on Health and Welfare (CSLCPHW)^20^. The statistical significance threshold was p<0.05 in this study.

## RESULTS

### Combustible cigarette use and intention to quit smoking

Table 1 shows the adjusted percentage (%) of combustible tobacco use and intention to quit smoking among current smokers. Of men without any diseases, 36.0% (n=487) currently smoked, while 33.0% (n=195) and 32.1% (n=80) of patients with one disease and more than two diseases, respectively, currently smoked. A significantly lower (p=0.045) prevalence of current smoking was observed in patients with cerebrovascular disease (24.3%) compared to those without any diseases (36.0%). Of patients with atopic dermatitis, 15.5% had no intention to quit smoking (this percentage was significantly lower than that of men without any diseases, 35.0%). On the other hand, 63.7% of patients with mental disorders had no intention to quit smoking (this percentage was significantly higher than that of men without any diseases).

**Table 1:** Adjusted percentages of combustible cigarette use and intention to quit smoking according to disease among Japanese people aged 40–69 years from a 2015 internet survey

	*n (%)*	*former n (%)*	*current smokers*		*No intention to quit smoking among current smokers*^2^	

			*n (%)*	*p^1^*	*n (%)*	*p^1^*
**Men**						
Total	2193.0	758.2 (34.6)	762.6 (34.8)		266.7 (35.0)	
**Disease numbers**						
0	1351.9 (61.6)	440.8 (32.6)	487.2 (36.0)	ref	170.5 (35.0)	ref
1	591.1 (27.0)	227.0 (38.4)	195.2 (33.0)	0.22	74.4 (38.1)	0.48
≧2	250.0 (11.4)	90.4 (36.2)	80.1 (32.1)	0.25	21.8 (27.2)	0.20
**Disease**						
Hypertension	572.1 (26.1)	217.2 (38.0)	187.7 (32.8)	0.19	52.1 (27.8)	0.07
Diabetes	247.7 (11.3)	90.1 (36.4)	80.2 (32.4)	0.28	19.6 (24.5)	0.08
Asthma	70.8 (3.2)	24.0 (33.9)	18.2 (25.7)	0.07	8.1 (44.7)	0.46
Atopic dermatitis	76.1 (3.5)	15.9 (20.9)	31.7 (41.6)	0.33	4.9 (15.5)	0.03
Cerebrovascular diseases	74.1 (3.4)	32.3 (43.6)	18.0 (24.3)	0.045	5.8 (32.5)	1.00
COPD	32.7 (1.5)	16.1 (49.2)	12.5 (38.2)	0.85	5.0 (40.0)	0.76
Cancer	39.8 (1.8)	13.3 (33.4)	10.5 (26.5)	0.32	2.7 (25.3)	0.75
Mental disorder	107.2 (4.9)	24.7 (23.1)	39.3 (36.7)	0.92	25.1 (63.7)	<0.01
depression	82.8 (3.8)	20 (24.6)	28 (33.9)	0.72	18.9 (67.5)	<0.01
other mental diseases	44.8 (2.0)	10 (22.5)	21 (46.1)	0.16	7.9 (38.2)	0.82
**Tobacco-caused diseases**						
Yes	432.7 (19.7)	159.3 (36.8)	138.6 (32.0)	0.15	60.5 (43.6)	0.06
**Women**						
Total	2239.0	347.4 (15.5)	217.1 (9.7)		65.4 (30.1)	
**Disease numbers**						
0	1572.8 (70.2)	244.5 (15.5)	151.0 (9.6)	ref	47.1 (31.2)	ref
1	509.0 (22.7)	76.5 (15.0)	50.0 (9.8)	0.86	17.4 (34.9)	0.73
≧2	157.2 (7.0)	26.5 (16.8)	16.1 (10.3)	0.78	0.8 (5.0)	0.04
**Disease**						
Hypertension	388.1 (17.3)	64.8 (16.7)	30.0 (7.7)	0.28	3.2 (10.7)	0.02
Diabetes	82.6 (3.7)	11.9 (14.4)	1.2 (1.5)	<0.01	- 3)	NA
Asthma	117.8 (5.3)	13.3 (11.3)	15.0 (12.7)	0.26	5.2 (34.9)	1.00
Atopic dermatitis	89.7 (4.0)	12.6 (14.1)	6.2 (6.9)	0.46	- 3)	NA
Cerebrovascular diseases	42.8 (1.9)	0.8 (1.9)	2.3 (5.3)	0.43	- 3)	NA
COPD	15.4 (0.7)	0.0 (0.0)	0.6 (3.8)	1.00	- 3)	NA
Cancer	49.7 (2.2)	7.2 (14.5)	3.1 (6.3)	0.62	- 3)	NA
Mental disorder	136.2 (6.1)	21.3 (15.6)	23.8 (17.5)	<0.01	8.4 (35.1)	0.81
depression	93.9 (4.2)	5.2 (5.5)	15 (16.1)	0.051	7.6 (50.3)	0.16
other mental diseases	80.7 (3.6)	17 (21.0)	12 (14.7)	0.13	1.1 (9.2)	0.11
**Tobacco-caused diseases**						
Yes	307.6 (13.7)	44.4 (14.5)	38.3 (12.5)	0.15	13.6 (35.5)	0.57

Cerebrovascular diseases include angina, cardiac infarct, and stroke. Cancer includes lung, oropharyngeal, larynx, esophagus, stomach, liver, pancreas, kidney, urinary tract, bladder cancer and others. Tobacco-caused diseases include diabetes, asthma, cerebrovascular diseases, COPD, cancers, and mental disorder^8^.

1) Fisher exact test 2) Analyzed for only current smokers 3) Data not shown due to low number of current smokers.

Among women, 9.6% (n=151) of those without any diseases currently smoked, while 9.8% (n=50) and 10.3% (n=16) of those with one disease or more than two diseases, respectively, currently smoked. Of patients with high blood pressure, 10.7% had no intention to quit smoking (this percentage was significantly lower than that of women without any diseases, 31.2%). Finally, 5.0% of patients with more than two diseases had no intention to quit smoking (this percentage was significantly lower than that of women without any diseases).

### Electronic and heat-not-burn cigarette use

Table 2 shows the adjusted percentage (%) of e-cigarette use. Of the men without any diseases, 7.7% (n = 105) had ever used e-cigarettes, while 7.7% (n=45) and 6.4% (n=16) of men with one disease or more than two diseases, respectively, had ever used e-cigarettes. Of the women without any diseases, 3.5% (n=56) had ever used e-cigarettes, while 2.1% (n=11) and 2.9% (n=4.6) of women with one disease or more than two diseases, respectively, had ever used e-cigarettes. Of the men without any diseases, 0.8% had ever used e-cigarettes in the past 30 days, while 0.9% and 0.4% of men with one disease or more than two diseases, respectively, had ever used e-cigarettes in the past 30 days (Table S5). Of the women without any diseases, 0.4% had ever used e-cigarettes in recent 30 days, while 0.5% and 0.0% of women with one disease or more than two diseases, respectively, had ever used e-cigarettes in recent 30 days (see Table S5 in the Supplementary file, where also data about dual use and comparison by smoking habits are given in Tables S6 and S7). Of current smokers without any diseases, 15.7% (n=100) had ever used e-cigarettes, while 11.5% (n=28) and 10.3% (n=10) of current smokers with one disease or more than two diseases, respectively, had ever used e-cigarettes (Table S7). Percentage of heat-not-burn tobacco use or dual use was very low (<0.1 %), regardless of sex and smoking status in 2015.

**Table 2:** Adjusted percentages of electronic and heat-not-burn cigarette use according to disease among Japanese people aged 40–69 years from a 2015 internet survey

	*n (%)*	*ever use*				*current use*			
		*e-cigarettes*		*heat-not-burn tobacco*		*e-cigarettes*		*heat-not-burn tobacco*	

		*n (%)*	*p^1^*	*n (%)*	*p^1^*	*n (%)*	*p^1^*	*n (%)*	*p^1^*
**Men**									
Total	2193.0	166 (7.6)		1.7 (0.08)		9.4 (0.4)		0.8 (0.04)	
**Disease numbers**									
0	1352 (61.6)	105 (7.7)	ref	1.2 (0.09)	ref	1.5 (0.1)	ref	0.3 (0.03)	ref
1	591 (27.0)	45 (7.7)	1.00	0.5 (0.08)	1.00	6.5 (1.1)	<0.01	0.5 (0.08)	1.00
≧2	250 (11.4)	16 (6.4)	0.52	0.0 (0.0)	NA	1.3 (0.5)	0.40	0.0 (0.0)	NA
**Disease**									
Hypertension	572 (26.1)	44 (7.7)	1.00	0.5 (0.08)	1.00	5.9 (1.0)	0.01	0.5 (0.08)	1.00
Diabetes	247 (11.3)	21 (8.6)	0.70	0.0 (0.0)	NA	0.0 (0.0)	NA	0.0 (0.0)	NA
Asthma	70 (3.2)	9.0 (13)	0.17	0.0 (0.0)	NA	0.9 (1.2)	0.14	0.0 (0.0)	NA
Atopic dermatitis	76 (3.5)	9.9 (13)	0.12	0.0 (0.0)	NA	0.9 (1.2)	0.15	0.0 (0.0)	NA
Cerebrovascular diseases	74 (3.4)	1.7 (2.3)	0.17	0.0 (0.0)	NA	0.4 (0.6)	1.00	0.0 (0.0)	NA
COPD	32 (1.5)	0.0 (0.0)	NA	0.0 (0.0)	NA	0.0 (0.0)	NA	0.0 (0.0)	NA
Cancers	39 (1.8)	2.0 (5.1)	0.76	0.0 (0.0)	NA	0.4 (1.1)	1.00	0.0 (0.0)	NA
Mental disorder	107 (4.9)	11 (9.9)	0.36	0.0 (0.0)	NA	1.5 (1.4)	0.20	0.0 (0.0)	NA
depression	83 (3.8)	7.1 (8.6)	0.83	0.0 (0.0)	NA	0.9 (1.1)	0.16	0.0 (0.0)	NA
other mental diseases	45 (2.0)	4.3 (9.7)	0.77	0.0 (0.0)	NA	1.5 (3.3)	0.09	0.0 (0.0)	NA
**Tobacco-caused diseases**									
Yes	433 (19.7)	29 (6.6)	0.53	0.0 (0.0)	NA	1.9 (0.4)	0.25	0.0 (0.0)	NA
**Women**									
Total	2239	71 (3.1)		0.6 (0.03)		2.6 (0.1)		0.0 (0.0)	
**Disease numbers**									
0	1573 (70.2)	56 (3.5)	ref	0.5 (0.03)	ref	0.007 (0.0004)	ref	0.0 (0.0)	ref
1	509 (22.7)	11 (2.1)	0.15	0.2 (0.03)	1.00	2.5 (0.5)	0.0595	0.0 (0.0)	NA
≧2	157 (7.0)	4.6 (2.9)	1.00	0.0 (0.00)	NA	0.0 (0.0)	NA	0.0 (0.0)	NA
**Disease**									
Hypertension	388 (17.3)	5.7 (1.5)	0.0502	0.0 (0.0)	NA	0.0 (0.0)	NA	0.0 (0.0)	NA
Diabetes	83 (3.7)	0.8 (1.0)	0.36	0.0 (0.0)	NA	0.0 (0.0)	NA	0.0 (0.0)	NA
Asthma	118 (5.3)	1.1 (0.9)	0.18	0.2 (0.15)	1.00	0.2 (0.2)	1.00	0.0 (0.0)	NA
Atopic dermatitis	90 (4.0)	0.0 (0.0)	NA	0.0 (0.0)	NA	0.0 (0.0)	NA	0.0 (0.0)	NA
Cerebrovascular diseases	43 (1.9)	0.3 (0.6)	0.4	0.0 (0.0)	NA	0.0 (0.0)	NA	0.0 (0.0)	NA
COPD	15 (0.7)	0.0 (0.0)	NA	0.0 (0.0)	NA	0.0 (0.0)	NA	0.0 (0.0)	NA
Cancer	50 (2.2)	5.5 (11)	0.01	0.0 (0.0)	NA	1.9 (3.9)	<0.01	0.0 (0.0)	NA
Mental disorder	136 (6.1)	7.2 (5.3)	0.34	0.0 (0.0)	NA	0.4 (0.3)	1.00	0.0 (0.0)	NA
depression	94 (4.2)	3.0 (3.2)	1.00	0.0 (0.0)	NA	0.4 (0.4)	1.00	0.0 (0.0)	NA
other mental diseases	81 (3.6)	5.0 (6.2)	0.22	0.0 (0.0)	NA	0.4 (0.5)	1.00	0.0 (0.0)	NA
**Tobacco-caused diseases**									
Yes	308 (13.7)	0.0 (0.0)	NA	0.0 (0.0)	NA	0.0 (0.0)	NA	0.0 (0.0)	NA

Cerebrovascular diseases include angina, cardiac infarct, and stroke. Cancer includes lung, oropharyngeal, larynx, esophagus, stomach, liver, pancreas, kidney, urinary tract, and bladder cancer. Tobacco-caused diseases include diabetes, asthma, cerebrovascular diseases, COPD, cancers, and mental disorder. 1) Fisher exact test.

## DISCUSSION

We did not find large differences in the use of electronic, heat-not-burn, and combustible cigarettes based on the number of diseases, except that the ‘no intention to quit smoking’ rate was lower in women with more than two diseases than in women without any diseases. On the other hand, differences in cigarette use were observed across diseases. This may be because some disease diagnoses (or treatments) do not motivate patients to quit smoking. For example, patients with mental disorders may be likely to continue smoking due to their belief that smoking relieves stress or anxiety. Actually, adult smoking in the US has declined by 55% in the general population, to 18%. However, comparable decreases in smoking rates did not occur among smokers with mental disorders^21^.

To our knowledge, this is the first study to examine electronic and heat-not-burn cigarette use among patients with a wide range of diseases, including mental disorders^6^. The differences in electronic and heat-not-burn cigarette use according to the diseases were not straightforward (Table 2). Previous studies mostly investigated the actual use of e-cigarettes among patients with cancer or asthma^6,8,9^. For example, 9.9% of adult cancer survivors had used e-cigarettes at least once in the previous month in the United States^9^. In the present study, the percentage of people who had used electronic or heat-not-burn cigarettes in the last 30 days was low in Japan: 0.9% for men with one disease, and 0.4% for men with more than two diseases (Table S3). However, because the Japanese market for electronic or heat-not-burn cigarettes has been steadily expanding since first introduced, we need to continue to monitor electronic or heat-not-burn cigarette use. In the US, current smokers new better and used more e-cigarettes than non-smokers^22,23^. Smokers believed that regular combustible cigarette products were more likely than e-cigarettes to cause lung cancer, oral cancer, and heart disease^24^. However, there is some controversy whether combustible tobacco is more harmful than e-cigarettes^25^. Therefore, further research is needed to evaluate the trends in electronic or heat-not-burn cigarette use, as well as the effect of electronic or heat-not-burn cigarettes on smoking cessation for patients with chronic diseases.

For combustible cigarette use, sex differences among chronic disease patients, especially in atopic dermatitis and mental disorders, were found. In all, 41.6% (n=32) of male atopic dermatitis patients currently smoked; this is the highest rate among all diseases. It is possible that many male current smokers with atopic dermatitis are not concerned about their disease and smoking. On the other hand, female patients might be concerned about the cosmetic aspects of atopic dermatitis^26^ and consider that if they stop smoking their condition will improve. In fact, active and passive exposure to smoke has been found to be associated with increased atopic dermatitis^27^. However, there are few studies about the relationship between smoking and atopic dermatitis. Both men and women with mental disorders had a high smoking rate and they did not quit. In the US, the percentages of current smoker with mental disorders (social phobia, posttraumatic stress disorder, major depression, bipolar disorder and so on) were high (35.9–68.8%) compared to those of the healthy control (22.5%)^28^. These data are consistent with our data, and this trend may be because some psychiatrists do not recommend smoking cessation strongly to their patients^27^, although the harmful effect of tobacco on mental disorders has been widely reported^20,29^. Psychiatrists have an important role to play in the recognition and treatment of cigarette use disorders among those with mental disorders^27^. It is important to encourage current smokers with mental disorders to quit smoking.

### Limitations

There are several limitations to the study. First, it is cross-sectional and self-reported, which makes it difficult to consider the result as a representative Japanese estimate or to establish a causal relationship between disease and tobacco use. Second, various diseases were analyzed, but the small numbers among some diseases, resulted in low statistical power. Third, due to the self-reported nature of the data, women may underreport tobacco use^30^.

## CONCLUSIONS

Differences in the use of electronic and combustible cigarettes according to the number of diseases were not obvious. Sex differences for smoking among chronic diseases patients, especially in atopic dermatitis and mental disorders, were found. As for atopic dermatitis, only male patients had the highest rate among all diseases. As for mental disorders, both male and female patients had a high smoking rate and they did not quit.
